# Derivation of a Human In Vivo Benchmark Dose for Perfluorooctanoic Acid From ToxCast In Vitro Concentration–Response Data Using a Computational Workflow for Probabilistic Quantitative In Vitro to In Vivo Extrapolation

**DOI:** 10.3389/fphar.2021.630457

**Published:** 2021-05-11

**Authors:** George Loizou, Kevin McNally, Jean-Lou C. M. Dorne, Alex Hogg

**Affiliations:** ^1^ Health and Safety Executive, Harpur Hill, Buxton, United Kingdom; ^2^ Scientific Committee and Emerging Risks Unit, European Food Safety Authority, Parma, Italy

**Keywords:** physiologically based kinetic, in silico, in vitro, reverse dosimetry, bayesian

## Abstract

A computational workflow which integrates physiologically based kinetic (PBK) modeling, global sensitivity analysis (GSA), approximate Bayesian computation (ABC), and Markov Chain Monte Carlo (MCMC) simulation was developed to facilitate quantitative *in vitro* to *in vivo* extrapolation (QIVIVE). The workflow accounts for parameter and model uncertainty within a computationally efficient framework. The workflow was tested using a human PBK model for perfluorooctanoic acid (PFOA) and high throughput screening (HTS) *in vitro* concentration–response data, determined in a human liver cell line, from the ToxCast/Tox21 database. *In vivo* benchmark doses (BMDs) for PFOA intake (ng/kg BW/day) and drinking water exposure concentrations (µg/L) were calculated from the *in vivo* dose responses and compared to intake values derived by the European Food Safety Authority (EFSA). The intake benchmark dose lower confidence limit (BMDL_5_) of 0.82 was similar to 0.86 ng/kg BW/day for altered serum cholesterol levels derived by EFSA, whereas the intake BMDL_5_ of 6.88 was six-fold higher than the value of 1.14 ng/kg BW/day for altered antibody titer also derived by the EFSA. Application of a chemical-specific adjustment factor (CSAF) of 1.4, allowing for inter-individual variability in kinetics, based on biological half-life, gave an intake BMDL_5_ of 0.59 for serum cholesterol and 4.91 (ng/kg BW/day), for decreased antibody titer, which were 0.69 and 4.31 the EFSA-derived values, respectively. The corresponding BMDL_5_ for drinking water concentrations, for estrogen receptor binding activation associated with breast cancer, pregnane X receptor binding associated with altered serum cholesterol levels, thyroid hormone receptor α binding leading to thyroid disease, and decreased antibody titer (pro-inflammation from cytokines) were 0.883, 0.139, 0.086, and 0.295 ng/ml, respectively, with application of no uncertainty factors. These concentrations are 5.7-, 36-, 58.5-, and 16.9-fold lower than the median measured drinking water level for the general US population which is approximately, 5 ng/ml.

## Introduction

In the environment of the modern world, chemicals of anthropogenic and natural origin are ubiquitous. The diversity of chemicals that risk assessors must appraise is large. For example, in the food sector this includes anthropogenic contaminants such as pesticides, biocides, food and feed additives, pharmaceuticals, air pollutants, persistent organic pollutants, heavy metals, perfluoroalkyl substances, brominated flame retardants, dioxins, and those of natural origin (marine biotoxins, mycotoxins, etc.) to name but a few. In this context, human risk assessment of chemicals aims to quantify exposures in human subpopulations from relevant sources and exposure routes (exposure assessment), to identify and characterize adverse effects and determine safe levels (hazard identification and characterization) as well as quantify risks associated with such exposures (risk characterization) (Ingenbleek et al., 2021).

Hazard identification and hazard characterization requires an understanding of what the human body does to the chemical, known as “toxicokinetics (TK)” and what the chemical does to the body, known as “toxicodynamics (TD).” Over the last century, the TD dimension and the derivation of acute and chronic safe levels of exposure for human health has been mostly addressed using animal toxicological studies in test species (rat, mouse, rabbit, and dog). Such studies allow the identification of an apical endpoint which is an observable outcome in the whole animal including clinical signs or pathologies indicative of a disease state that can result from exposure to a toxicant. For a given chemical, the key apical endpoint is identified based on the role of the endpoint in causing adversity and the dose-response relationship observed in the critical toxicity study in a test species (rat, mouse, rabbit, and dog). This principle is based on the requirement that all relevant adverse effects should be considered in order to achieve a sufficient level of protection. As a consequence, all reliable toxicological studies and relevant apical effects are considered to identify the highest dose that does not produce any statistically significant increase in the incidence of an adverse effect. Such a dose or concentration, known as the reference point (RP) or point of departure (PoD), is defined as the point on a toxicological dose–response curve established from experimental data that corresponds to an estimated no-observed-adverse-effect level (NOAEL) or low effect level. PoDs are used as the basis for the derivation of safe levels of human exposure known as health-based guidance values (HBGVs) (Ingenbleeks et al., 2021).

The most common RPs or PoDs are the NOAEL and the benchmark dose (BMD). The NOAEL approach uses statistical methods to identify the tested dose with no significant effect compared to the control group. The BMD approach fits a dose–response model(s) to a complete dose–response dataset to identify the benchmark dose lower confidence limit (BMDL) for a selected observed level of effect, the benchmark response (BMR) (e.g., a 10% response). The BMD is increasingly preferred by regulatory agencies, but its use is often limited by test design (Bokkers and Slob, 2005; Bokkers and Slob, 2007; European Food Safety, 2009; EFSA Scientific Committee et al., 2017). From the RP or PoD, HBGVs are derived most often by applying a default uncertainty factor (UF) of 100 allowing for interspecies (10-fold) and inter-individual differences (10-fold) when no TK and TD information is available. Alternatively, when chemical-specific or pathway-specific TK or TD information exists, more informed UF values can be used. For chemicals with a threshold for toxicity (non-genotoxic) chemicals, HBGVs in the food and feed safety area include the acceptable daily intake (ADI) for food additives, feed additives and pesticides, tolerable daily intake (TDI) for contaminants, upper limits (UL) for vitamins and minerals, and, for acute effects, the acute reference dose (ARfD) (Sheffer, 2009; EFSA Scientific Committee, 2012).

Since PoDs and HBGVs mostly rely on toxicological studies in test species, the international scientific community has been involved in considerable research and validation efforts to reduce animal testing and provide alternative-to-animal testing methods (i.e., *in vitro* and *in silico*) known as new approach methods (NAMs). The development of an alternative to animal human safety testing strategy for chemicals has been described as being akin to seeking the Holy Grail (Louisse et al., 2016). Efforts toward achieving this goal have been ongoing since publication of the United States National Research Council (NRC) report “Toxicity Testing in the 21st Century: A Vision and a Strategy” (NRC, 2007). In Europe, the development of such a strategy received impetus following the full marketing ban enforced under the EU Cosmetics Regulation (EC 1223/2009) in 2013 for cosmetic products and ingredients tested on animals anywhere in the world (Coecke et al., 2013).

In practice, alternative to animal methods invariably refer to an *in vitro* bioassay-based strategy that ideally uses human cell lines primarily for the determination of a RP. Importantly, *in vitro* concentration–response data must be converted to *in vivo* dose responses in order to be used in human safety testing of chemicals. This activity is known as quantitative *in vitro* to *in vivo* extrapolation (QIVIVE) (Bale et al., 2014; Hartung, 2017). Examples of QIVIVE increasingly involve the application of physiologically based kinetic (PBK) modeling–based reverse dosimetry for the translation of *in vitro* to *in vivo* responses and the derivation of *in vivo* BMDs (Adam et al., 2019; Boonpawa et al., 2017; Li et al., 2017; Louisse et al., 2016; Louisse et al., 2010; Louisse et al., 2012; Punt et al., 2017; Shi et al., 2020; Strikwold et al., 2017a; Strikwold et al., 2013; Strikwold et al., 2017b; Zhang et al., 2020; Zhao et al., 2019). Within this approach, all parameters, other than input dose or exposure, are held fixed at central values. An optimization routine is implemented in order to minimize the discrepancy between a target *in vivo* concentration, predicted by the PBK model and a given *in vitro* concentration. The dose concentration which corresponds to the target *in vitro* concentration is considered to be a surrogate for the *in vivo* concentration. However, these studies did not account for PBK model structure or parameter uncertainty. Therefore, an algorithm, described in detail previously (McNally et al., 2018), was developed to extrapolate *in vitro* concentration–response to *in vivo* dose–response relationships while applying a rigorous statistical framework for accommodating uncertainty in both the parameters of the PBK model, the quality of fit of the model to measure biological monitoring data, and a consideration of how this affects an *in vivo* dose–response relationship in the context of QIVIVE (Judson et al., 2011). This is important since the level of detail (fidelity) captured in the model could have a bearing on model output (Rowland et al., 2017). Understanding and quantifying the level of uncertainty in each step of a chemical safety assessment with NAMs is important for the development of confidence in this approach (Berggren et al., 2017). The workflow uses global sensitivity analysis (GSA), PBK modeling, approximate Bayesian computation (ABC), and Markov Chain Monte Carlo simulation to convert *in vitro* concentration–response data to *in vivo* dose–response data (McNally et al., 2018).

There are a number of advantages with regard to exposure or dose reconstruction provided by this probabilistic approach. First, defining informative prior distributions around parameters converts a deterministic model to a population model which can account for inter-individual variability. Second, the probabilistic approach is appropriate for systems where tissue dose is not necessarily linearly related to external exposure. Finally, this combination can extract population variability and multiple routes of exposure information integrated within pharmacokinetic data (McNally et al., 2012; McNally et al., 2018).

In this report, we tested the workflow using HTS *in vitro* concentration–response data for perfluorooctanoic acid (PFOA). The data were obtained from the ToxCast/Tox21 database on the US EPA Chemistry Dashboard (Williams et al., 2017) and translated to *in vivo* dose responses with a PBK model for PFOA (Worley et al., 2017). An *in vivo* BMD for PFOA intake (ng/kg BW/day) was calculated from the *in vivo* dose responses and compared to the intake also derived from a BMD used by the European Food Safety Authority (EFSA) in the scientific opinion on the risk to human health related to the presence of perfluoroalkyl substances in food for effects on the immune system (EFSA Contam Panel, 2020) and previously for increases in serum cholesterol (EFSA Contam Panel et al., 2018).

PFOA is a synthetic chemical comprised of a fully fluorinated eight carbon chain with a carboxylic acid functional group. It was used in the manufacture of many consumer products including fast food wrappers, non-stick cookware, a stain-resistant coating used on carpets and other fabrics (Bartell et al., 2010). PFOA is both hydrophobic and lipophobic, does not break down in the environment, contaminates drinking water sources, and accumulates in food chains (Bartell et al., 2010; Rayne and Forest, 2009; Shin et al., 2011). It is not metabolized in the human body, and the half-life is estimated to be between four and five years (Emmett et al., 2006; Steenland et al., 2010). Epidemiological studies support a possible association with liver, pancreatic, and testicular cancer (Lau et al., 2007; Barry et al., 2013; Vieira et al., 2013) and breast cancer (Bonefeld-Jorgensen et al., 2011).

The purpose of this study, however, was not to propose an animal-free risk assessment for PFOA, since it is recognized that much work is still needed to demonstrate *in vitro* to *in vivo* concordance for systemic, chronic exposures to environmental xenobiotics. Instead, this study serves to further demonstrate the utility of the algorithm in anticipation of the acceptance of *in vitro* concentration–response data in chemical risk assessment.

Nevertheless, a workflow was followed which would be similar to most risk assessment approaches. For example, epidemiological studies reported that PFOA is a suspected endocrine disruptor (Pierozan et al., 2018) associated with a risk of breast cancer (Bonefeld-Jorgensen et al., 2011). Other studies observed an increase in cellular triglyceride levels and *in vivo* expression of genes involved in cholesterol metabolism (Fletcher et al., 2013) and gene sets related to “PPAR signaling,” “lipid metabolism,” “fatty acid beta oxidation,” and “tRNA amino-acylation” which are related to “cholesterol biosynthesis” which may be associated with hepatic steatosis (Louisse et al., 2020). Functional thyroid disease was observed in a large cohort of people exposed to PFOA in drinking water contaminated from a mid-Ohio River Valley chemical plant (Winquist and Steenland, 2014) where reduced expression of parathyroid hormone 2 receptor which may increase risk for conditions related to parathyroid hormone signaling was also observed (Fletcher et al., 2013; Galloway et al., 2015). Finally, recent epidemiological studies found associations with effects on the immune system (reduced antibody titers) (EFSA Contam Panel, 2020) which were hypothesized to result from a dysregulated cytokine/chemokine response and impaired neutralizing antibody response (Lee et al., 2017). Therefore, *in vitro* datasets were selected as measured responses that could be related to the mechanistic understanding related to PFOA toxicity and observations in human exposure studies, such as, estrogen receptor binding activation associated with breast cancer, pregnane X receptor binding associated with hepatic steatosis, thyroid hormone receptor *α* binding leading to thyroid disease, and immunotoxicity (pro-inflammation from cytokines).

## Materials and Methods

### PBK Model

#### Software

The generic PBK model code describing the kinetics of perfluorooctanoic acid (Worley et al., 2017) was provided by Dr Rachel Worley^1^ in CSL syntax, the equation-based language implemented in acslX™ software. However, support for acslX™ was discontinued in November 2015 (Lin et al., 2017). Therefore, the CSL code was translated into the GNU MCSim language (version 6.1.0.)^2^ and run under Windows 7 using RStudio (RStudio Team, 2016). Files for running MCSim under windows, tools and instructions for installation are available from Github^3^.

In order to perform probabilistic simulations the model code was further modified to ensure that logical constraints on mass balance and blood flow to the tissues were met by adopting the re-parameterizations described by Gelman et al. (1996).

The PBK model was evaluated using RVis, an open access PBK modeling platform^4^ which provides an intuitive user-friendly interface with which to interact with MCSim and the R platform^5^. The model equations were solved using MCSim which writes an output file in tab separated values (TSV) format which is then input into the R environment and read by the R packages required for the various analyses. Global sensitivity analysis (GSA) of model outputs (Morris screening test and extended Fourier Amplitude Sensitivity Test (eFAST) were conducted using the Sensitivity package of R. Reshaping of data and plotting was done using the reshape and ggplot2 packages, respectively (Wickham, 2007; Pouillot and Delignette-Muller, 2010; Soetaert et al., 2010; Pujol and Iooss, 2015). The main effects and total effects (McNally et al., 2011) were computed at each time point, and parameter sensitivities were studied over this period using Lowry plots generated as described in McNally et al. (2011).

Benchmark dose values (BMDs) were calculated using PROASTweb version 67.0^6^ and R version 3.4.3^7^. All plots were created using R and ggplot2 (R Development Core Team, 2008; Wickham, 2016).

### Hardware

The computer used in this study was a Dell Optiplex 9,020 with an Intel(R) Core™ i5-4590 CPU@3.30 GHz with 8.00 GB RAM running Windows 7 Enterprise Service Pack 1. To run the computationally intensive simulations for QIVIVE, work was transferred to specially provisioned cloud infrastructure. Specifically, hardware was allocated using Microsoft Azure IaaS (infrastructure as a service). The specification was the F-series (F8s_V2), which is compute-optimized and suitable for running applications. The Fs v2 series is hyper-threaded and based on the 2.7 GHz Intel Xeon^®^ Platinum 8,168 (SkyLake) processor. Onto this hardware were installed Ubuntu Server 18, GNOME desktop, R, required R packages, and RStudio. Remote access was enabled using xrdp^8^.

### Workflow

*In vivo* serum concentrations (CA) at steady state were simulated in order to calibrate and evaluate model performance against the human biological monitoring data used by Worley et al. (2017). *In vivo* hepatic tissue PFOA concentrations (CL) were predicted during QIVIVE because HepG2 cells are derived from the human liver and are considered to be an *in vitro* surrogate for the liver *in vivo*.

The key to the approach described below is the recognition that the PBK model is an imperfect approximation to reality. Exact matching of the chosen PBK model response to an *in vitro* concentration suggests a higher degree of belief in the model than is warranted and is thus not desirable. By accepting a discrepancy between the two, within a specified threshold, model uncertainty is thus accommodated and an error term is created that can be exploited by efficient sampling techniques. The workflow described in McNally et al. (2018) was thus followed.

The modeling framework comprised five steps as described in McNally et al. (2018):

1. Probability distributions for model parameters were specified (see Table 1 for list of model parameters). In addition to the parameter distributions used by Worley et al. (2017) (shown in italics) in Table 2, distributions for the remaining parameters were derived from various sources. Distributions for VKC, QCC, QLC, and QKC were obtained from PopGen by generating a virtual healthy cohort of 50% male, 50% female Caucasian, black and nonblack Hispanic people (McNally et al., 2014); VplasC and Htc were derived from Crews et al. (2009) and ICRP (2002), whereas for the remaining parameters for which no information was readily available, uniform distributions were ascribed based on physiologically feasible estimates. These were VPTCC, PK, PR, Vmax_baso_intro, KM_baso, kdiff, kabsc, kunabsc, GEC, K0C, and kefflux.
2. Morris screening and eFAST of PFOA CA and CL were conducted at steady state. This required simulation of an exposure period of 120,100 h (13.71 years) in order to encompass the target time interval once steady state was achieved. The target time interval is a period of exposure over which the average of the dose metric was estimated in order to account for the variations caused by four, 15 min drinking events per day. The area under the curve (AUC) for CA and CL concentrations over a target time interval from 100,000 to 120,000 h was simulated.
3. The top ranked parameters from the Morris screening were further examined using a variance-based sensitivity analysis using eFAST–the insensitive parameters determined by the Morris test were held fixed at default values in this second phase of sensitivity analysis.
4. Refinement of the parameter ranges through calibration using the blood biomonitoring data of (ATSDR, 2016) and (Bartell et al., 2010). A statistical model was specified to link predicted concentrations in serum to biomonitoring data. A log-normal error model was assumed with calibration achieved using Markov Chain Monte Carlo implemented in GNU MCSim.
5. Estimation of a distribution of the daily drinking water concentration (Intake) and drinking water exposure concentrations (ExposedDW), corresponding to each of seven or four experimental *in vitro* concentrations (see Supplementary Table S1) while accounting for model and parameter value uncertainty. This was achieved using a two-step approximate Bayesian computation (ABC) approach. In the first phase, 500 parameter sets were drawn for sensitive parameters from uniform distributions based upon the refined limits resulting from calibration. These were paired with samples drawn for Intake and ExposedDW. The PBK model was run for each of these 500 parameter sets. The parameter sets that corresponded to predictions of CL or CA within ±7.5% of the target *in vitro* concentration were retained and the covariance matrix of the parameters calculated. In the second phase, a more efficient parameter space search was conducted using ABC MCMC. A proposed move was accepted if within ±5% of the target concentration. Four chains were run, each for 2,500 iterations. The above approach was repeated for each of the seven or four dose concentrations.

**TABLE 1:** Model parameters.

Physiological parameter	Abbreviation	Kidney transport parameters (contd)	Abbreviation
Body weight	BW	Basolateral transporter relative activity factor	RAFbaso
		Glomerular filtration rate	GFRC
Tissue volumes (fraction of BW)		Proximal tubule cell protein content	Protein
Liver	VLC		
Kidney filtrate	VfilC	**Rate constants**	
Kidney	VKC	Biliary elimination	KbileC
Plasma	VplasC	Urinary elimination	KurineC
Proximal tubule cells	VPTCC		
		Diffusion from proximal tubule cells	kdif
Cardiac output (CO)	QCC	Small intestine to liver absorption	kabsc
Blood flows (fraction CO)		Fecal elimination	kunabsc
Liver	QLC	Gastric emptying	GEC
Kidney	QKC	Stomach to liver absorption	K0C
Hematocrit	Htc		keffluxc
		Daily urine volume production	Kvoid
Chemical-specific parameters		**Exposure parameters**	
Plasma unbound fraction	Free	Drinking water concentration	backgrounddw
Tissue: blood partition coefficients		Contaminated drinking water concentration	ExposedDW
Liver	PL	Daily drinking water consumption	DWtotal
Kidney	PK	Past nondrinking water ingestion rate	Ingest_past
Rest of body	PR	Current nondrinking water ingestion rate	Ingest_current
		Total number of drinks per day	Drinks
Kidney transport parameters		Drinking event time	Tlendw
*In vitro* apical transporter maximum velocity uptake rate	Vmax_apical_in vitro	Duration of exposure	tbackground
*In vitro* apical transporter Michaelis–Menten constant	KM_apical		
Apical transporter relative activity factor	RAFapi		
*In vitro* basolateral transporter maximum velocity uptake rate	Vmax_baso_in vitro		
*In vitro* basolateral transporter Michaelis–Menten constant	KM_baso		

**TABLE 2:** Parameter distributions used in Morris screening, global sensitivity analysis, approximate Bayesian computation, and Markov Chain Monte Carlo simulation.

Parameter	Unit	Mean	SD	Lower bound	Upper bound	Distribution
Physiological						
BW^a^	Kg	4.36	0.313	3.747	4.973	Lognormal
GFRC	L/h/Kg kidney	3.14	0.294	2.564	3.716	Lognormal
Protein	mg protein/proximal tubule cell	2 × 10^–6^	6 × 10^–7^	8.24 × 10^–7^	3.18 × 10^–6^	Normal
Tissue volumes						
VLC	L/Kg BW	0.026	0.0078	0.0107	0.0413	Normal
VfilC	L/Kg BW	0.0004	0.00012	0.000165	0.000635	Normal
VKC	L/Kg BW	0.004	0.0008	0.0024	0.0056	Normal
VplasC	L/Kg BW	0.0428	0.009	0.025	0.061	Normal
VPTCC	L/g kidney	1.35 × 10^–4^	-	7.7 × 10^–5^	1.9 × 10^–4^	Uniform
Cardiac output (CO)						
QCC	L/h/kg BW^0.75^	12.5	2	8.5	16	Normal
Blood flows (fraction CO)						
QLC	Unit less	0.25	0.05	0.15	0.35	Normal
QKC	Unit less	0.175	0.03	0.12	0.24	Normal
Htc	Unit less	0.44	0.09	0.26	0.62	Normal
Chemical-specific parameters						
PL	Unit less	0.01	0.198	−0.378	0.398	Lognormal
PK	Unit less	1.17	0.2	0.77	1.6	Normal
PR	Unit less	0.11	0.02	0.07	0.15	Normal
Vmax_apical_invitro	pmol/mg protein/min	10.48	0.325	9.843	11.117	Lognormal
KM_apical	µg/L	11.25	0.161	10.929	11.561	Lognormal
RAFapi	Unit less	−7.31	0.294	−7.886	−6.734	Lognormal
KbileC	/h/Kg^−0.25^	−9.25	0.294	−9.826	−8.674	Lognormal
KurineC	/h/Kg^−0.25^	−2.81	0.294	−3.386	−2.243	Lognormal
Free	Unit less	−3.96	0.294	−4.536	−3.384	Lognormal
Vmax_baso_in vitro	pmol/mg protein/min	439.2	90	259.2	619.2	Normal
KM_baso	µg/L	20,100.0	4,000	12,100	28,100	Normal
RAFbaso	Unit less	1.0	0.2	0.6	1.4	Normal
Rate constants						
Kdif	L/h	0.001	0.0002	0.0006	0.0014	Normal
Kabsc	1/(h×BW^−0.25^)	2.12	0.04	1.3	2.9	Normal
Kunabsc	1/(h×BW^−0.25^)	7.06 × 10^–5^	1.0 × 10^–5^	5.1 × 10^–5^	9.1 × 10^–5^	Normal
GEC	1/(h×BW^−0.25^)	3.5	0.7	2.1	4.9	Normal
K0C	1/(h×BW^−0.25^)	1.0	0.2	0.6	1.4	Normal
Keffluxc	1/(h×BW^−0.25^)	0.1	0.02	0.06	0.14	Normal
Exposure parameters						
ExposedDW	µg/L	1.22	0.294	0.648	1.800	Lognormal
DWtotal	L/day	0.181	0.503	-0.805	1.167	Lognormal
Ingest_past	µg/h	−3.69	0.294	−5.570	−3.270	Lognormal
Ingest_current	µg/h	−4.65	0.294	−5.226	−4.074	Lognormal

a Italicized parameter distributions were taken directly from Worley et al. (2017). For the remaining parameter distributions where only point values were reported (in supplementary materials of Worley et al. 2017), best estimate standard deviations and normal distributions were ascribed.

Finally, a PoD, taken to be the benchmark dose (BMDL_5_) lower bound in the *in vivo* dose response relationship, was estimated.

### *In vitro* Data

*In vitro* concentration–response data were obtained from the ToxCast/Tox21 database available from the Bioactivity section of the United States Environmental Protection Agency Chemistry Dashboard^9^. ToxCast was created as a screening program which is reflected in the assay design. The purpose was to maximize throughput, minimize false negatives, and facilitate data processing for computational exercises and modeling to identify patterns^10^. ToxCast was not designed for the identification of molecular initiating events (MIEs) in the development of adverse outcome pathways (AOPs). Appropriate datasets were obtained by first filtering to retain only those that were active (positive hit-call), that is, had an AC_50_ (concentration at which 50% maximum activity was observed) derived from the Hill or Gain-Loss model where both the modeled and observed maximum responses met or exceeded an efficacy cutoff (Filer et al., 2017) and had no warning signs (flags). However, two datasets with flags indicating possible unwanted influence were retained because the shape of the response curve was similar to the curves for the other assays. All datasets were conducted using a human cell line. Datasets with an associated AOP and with the lowest AC_50_ value were selected. The rationale adopted was analogous to the process followed by regulatory agencies where generally, a NOAEL or BMD value is identified and used for the safety assessment of any given chemical. However, very few datasets had an associated AOP. Two of the four selected datasets had AOPs:

1. Estrogen receptor binding (assay name: ATG_ERE_CIS_up, AC50 = 33.82 µM). No flags. AOP estrogen receptor activation associated with breast cancer^11^
2. Pregnane X receptor binding (assay name: ATG_PXRE_CIS_up, AC50 = 35.28 µM). No flags. AOP inhibition, mitochondrial fatty acid *ß*-oxidation associated with hepatic steatosis^12^
3. Thyroid hormone receptor binding (assay name: ATG_THRa1_TRANS_dn, AC50 = 22.33 µM). Flag: hit-call potentially confounded by overfitting.
4. Plasminogen activation, urokinase receptor protein (assay name: BSK_3C_uPAR_down, AC50 = 1.63 µM). Flag: <50% efficacy, hit-call potentially confounded by overfitting.

Assays 1–3 were conducted in 24-well plates using human liver HepG2 cells and dimethyl sulfoxide as dilution solvent. Assays 1 and 2 generate a profile of transcription factors (TFs) in eukaryotic cell activities that represent a stable and sustained cell signature that clearly distinguishes different cell types, subcellular biochemical perturbations reflected in specific gene regulatory network alterations, and ultimately pathological conditions (Romanov et al., 2008; Martin et al., 2010). For example, endocrine disrupting chemicals (EDCs), particularly estrogen receptor (ER) agonists, are thought to contribute to birth defects and the incidence of breast cancer (Judson et al., 2015; Judson et al., 2017). Assay 3 measures inducible changes in human thyroid hormone receptor alpha (GAL4-THRa). Assay 4 is an immunotoxicity endpoint conducted on umbilical vein endothelium human vascular primary cells in 96-well plates and measured urokinase receptor protein, where changes in protein expression were conditioned to simulate pro-inflammation from cytokines (Houck et al., 2009; Kleinstreuer et al., 2014).

The original *in vitro* concentrations downloaded from the Chemistry Dashboard were expressed as Log_10_ μM and the corresponding responses as Log_2_ fold induction (Supplementary Table S1). The concentration and response data were converted to the natural scale in order to be used in the ABC algorithm. Also, concentrations were expressed in µg/L or ng/ml for direct comparison with the biological monitoring data from individuals whose primary drinking water source was the West Morgan East Lawrence Water Authority in North Alabama (ATSDR, 2016), the Little Hocking Water Association in the Mid-Ohio River Valley, and the Lubeck Public Service District in West Virginia (Bartell et al., 2010).

The original *in vitro* concentrations were considered to be nominal (applied) concentrations. Therefore, in order to estimate, to some extent, the bioavailable (free) concentration of PFOA in the *in vitro* assays assuming differential partitioning (to protein, lipid, and plastic) a simple approach using the Log_10_ of the octanol/water partition coefficient (Log P_o:w_) for PFOA was applied. According to Proença et al. (2019), pyrene (Log P_o:w_ 4.88–4.93) was predicted to have 2.3% of unbound (bioavailable) chemical relative to the nominal amount. Therefore, PFOA (Log P_o:w_ = 4.8–4.9) was predicted to have a similar *in vitro* bioavailability. The final *in vitro* concentrations input into the ABC algorithm were 2.3% of the nominal concentrations (Supplementary Table S1). However, a further caveat is that this is an estimate which ignores the effect of water solubility and pKa on the differential partitioning of PFOA.

### Calculation of *In Vivo* Benchmark Dose

**FIGURE 1:**
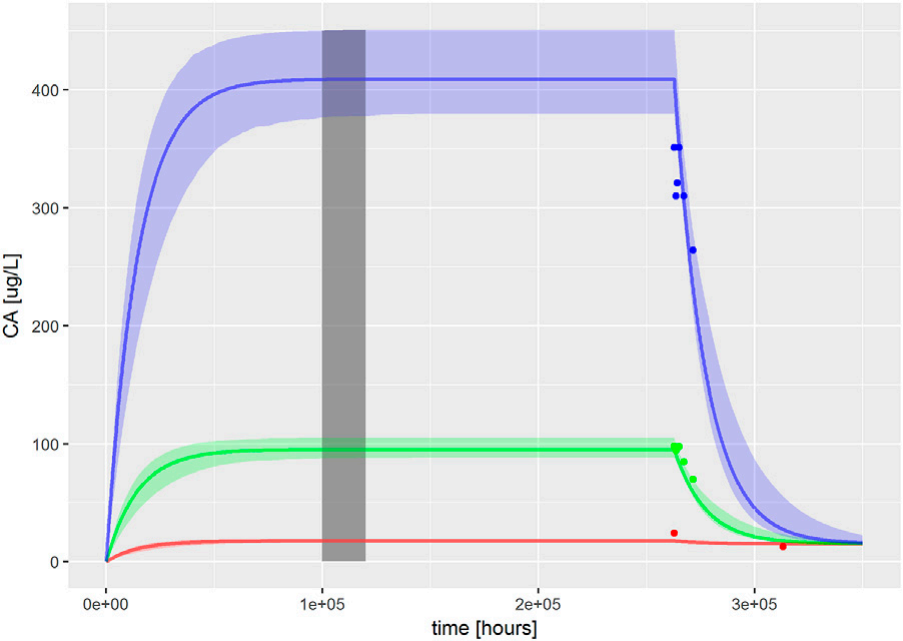
PBK model for PFOA was evaluated by reproducing Figures 2–4 from Worley et al. (2017). The solid, colored lines represent simulations of ExposedDW (drinking water PFOA concentrations) which were set to the highest concentration reported for each water authority; these were 0.04 μg/L (red), 1.0 μg/L (green), and 4.9 μg/L (blue) for North Alabama, Lubeck Public Service District, and Little Hocking Water Association, respectively. The simulations were for 30 years with a further 10-year postexposure period. The corresponding serum PFOA concentrations are shown as solid colored symbols. The vertical gray shaded band between 100,000 and 120,000 h (11.4–13.7 years) highlights the point where steady state was judged to have been achieved; the model predictions from this period were used for QIVIVE calculations.

The dose–response curves were predicted by simulating four, 15 min drinking events per day, over a target time interval of 100,000–120,000 h (a period over which steady state was reached) (Figure 1). The mode 2.5 and 97.5% of the credible interval values were calculated for the most sensitive parameters identified by GSA. BMD values were determined for ExposedDW, the contaminated drinking water concentration, and Intake the daily drinking water concentration, estimated for each *in vitro* concentration. Intake, with units of ng/kg BW/day, was derived in order to make direct comparisons with BMD values used in risk assessments. Intake was calculated as follows:

$$\text{Intake}=\frac{\text{ExposedDW}\times \text{DWtotal}}{\text{BW}},$$ (1)

where ExposedDW, (µg/L), DWtotal, the daily drinking water consumption (L) and BW, is body weight (kg).

Each dataset of seven *in vivo* CL or four CA mode concentrations and corresponding fold inductions was uploaded to PROASTWeb which fitted six candidate models that were suitable for continuous (value for each individual) response data. The benchmark dose 5% lower bound corresponding to the most conservative model that provided an adequate fit (as assessed by the software) to the data was determined. A benchmark response of 0.05 (5%) was specified.

## Results

### Morris Screening

Due to the stochastic nature of the Morris test parameter, rankings were derived by identifying the mode for each parameter over six simulations. From the entire set of 33 parameters studied using the Morris test, the model output (CL and CA) was judged to be insensitive to 20 parameters; these were held fixed at default values in the second phase of sensitivity analysis. Thirteen parameters (ExposeDW, DWtotal, BW, Vmax_apical_in vitro, RAFapi, Free, GFRC, kurinec, protein, VfilC, kbilec, PL, and VLC) were further studied in a variance-based sensitivity analysis using the eFAST technique.

### eFAST

**FIGURE 2:**
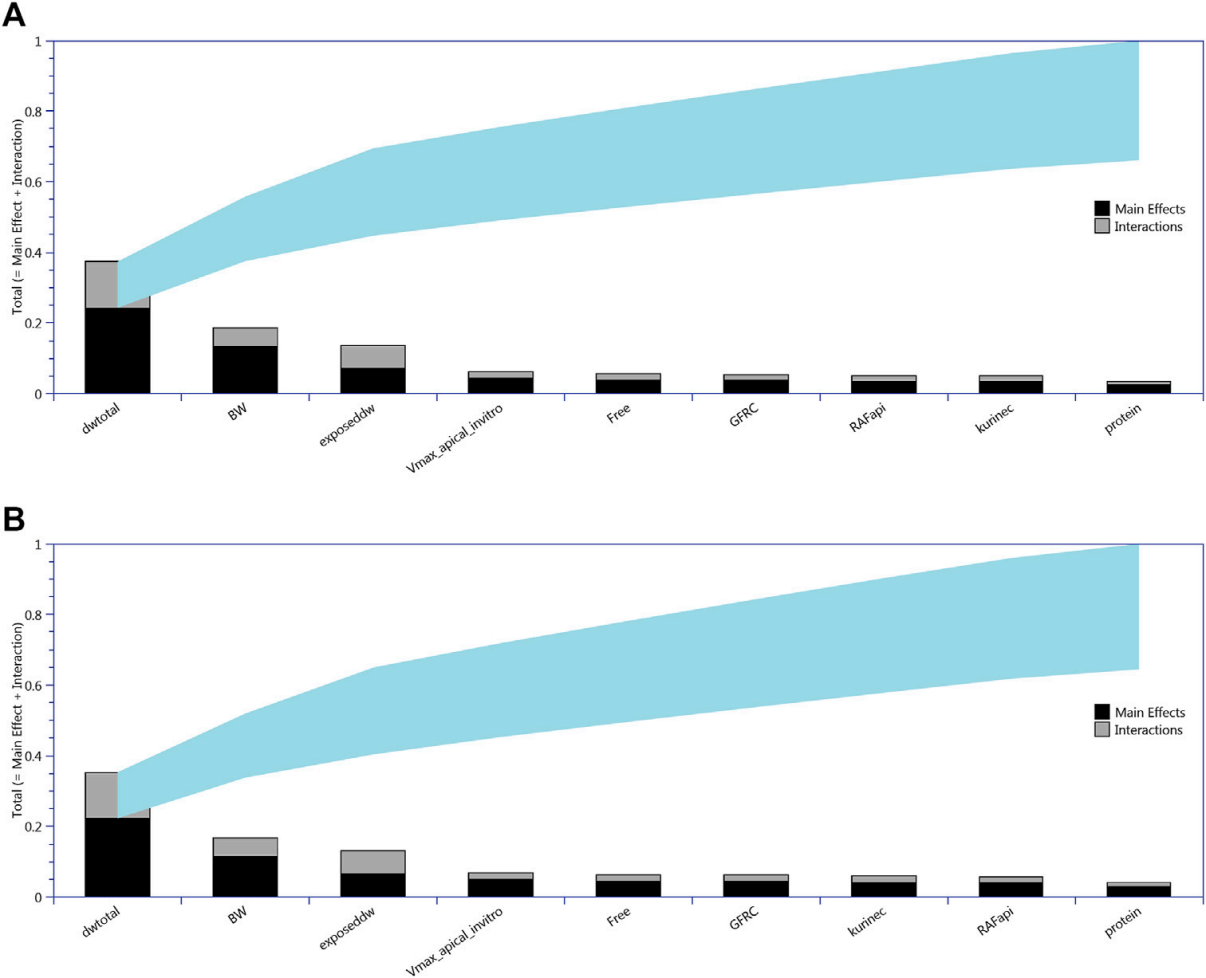
Lowry plots of the eFAST quantitative measure of the most sensitive parameters identified by Morris screening following oral (drinking water) exposure. The total effect of a parameter S_Ti_ comprised the main effect S_i_ (black bar) and any interactions with other parameters (gray bar) given as a proportion of variance. The ribbon, representing variance due to parameter interactions, is bounded by the cumulative sum of the main effects (lower bound of ribbon) and the minimum of the cumulative sum of the total effects (upper bound of ribbon). **(A)** For CL, liver cell concentrations **(upper panel)** and **(B)** for CA, serum concentrations **(lower panel)**.

All 13 parameters accounted for 100% variance in CL and CA (Figure 2). However, VLC, PL, kbilec, and VfilC made a total contribution of 10–11% to the overall variance over the target time interval. Therefore, in order to reduce computational overhead, they were held fixed at default values in onward analysis. The following parameters were included for model calibration and parameter estimation by the algorithm; ExposedDW, DWtotal, BW, Free, GFRC, kurinec, protein, RAFapi, and Vmax_apical_in vitro.

### Refinement of Exposure Assessment and Derivation of Chemical-Specific Adjustment Factor for Perfluorooctanoic Acid

The PBK model for PFOA was evaluated by reproducing Figures 2–4 from Worley et al. (2017). Sensitive parameters were subsequently calibrated, as described in methods, based upon ExposedDW set to the highest concentration reported for each water authority, these were 0.04, 1.0, and 4.9 μg/L for North Alabama (ATSDR, 2016), Lubeck Public Service District, and Little Hocking Water Association (Bartell et al., 2010), respectively.

**FIGURE 3:**
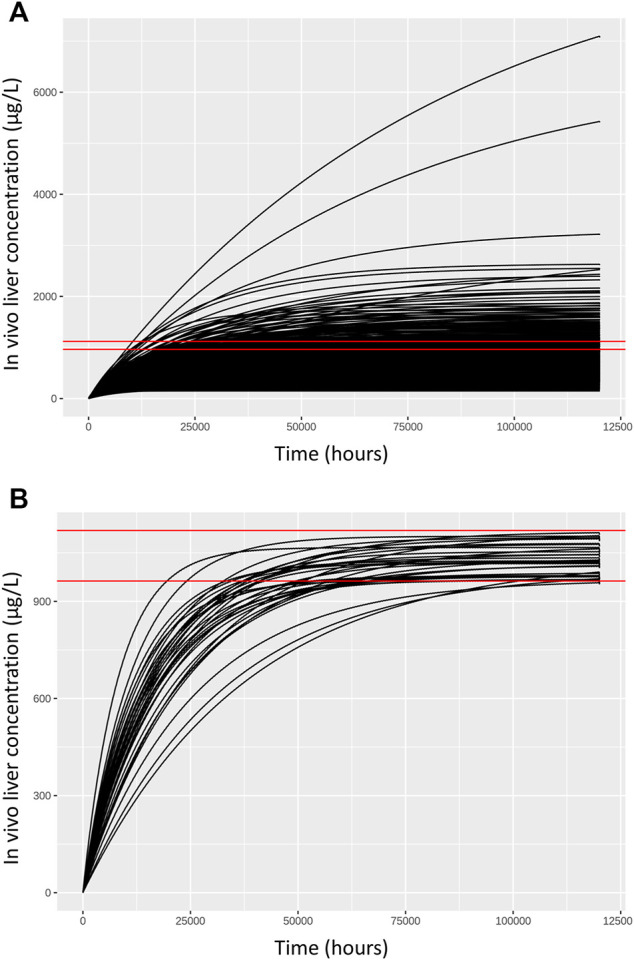
Comparisons of concentration response profiles simulated in the rejection phase were run for each dose concentration. A typical example is shown for a target concentration of 1,035.175 μg/L. **(A)** 500 concentration response profiles following 120,000 h exposure (upper panel) and **(B)** retained samples within a relative error of 7.5% (lower panel).

**FIGURE 4:**
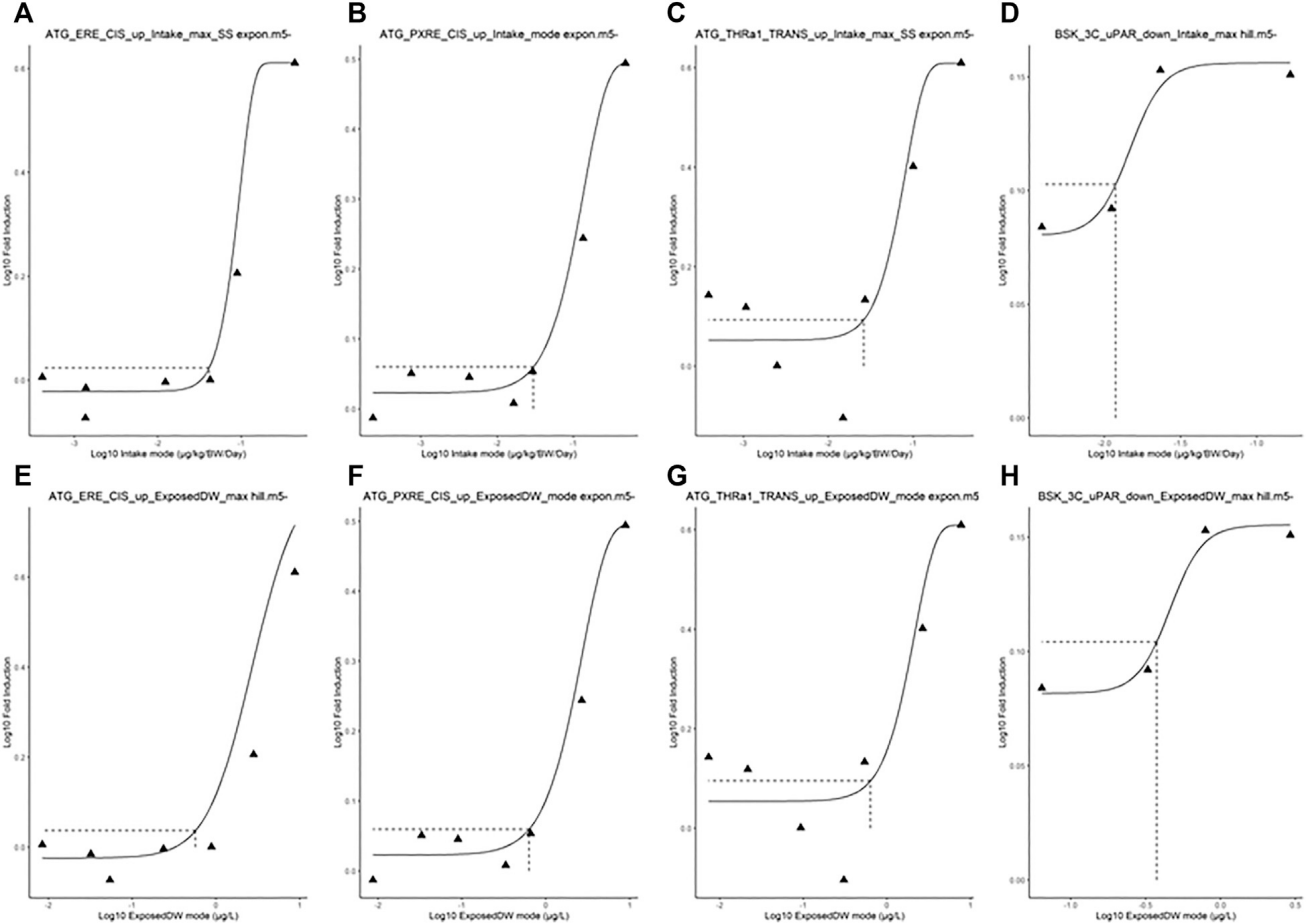
Typical predicted *in vivo* dose–response curves for Intake **(upper panels)** and ExposedDW **(lower panels)** for each of the *in vitro* datasets. These were for estrogen receptor binding activation leading to breast cancer **(A)** and **(E)**, pregnane X receptor binding leading to hepatic steatosis **(B)** and **(F)**, thyroid hormone receptor *a* binding leading to thyroid disease **(C)** and **(G),** and immunotoxicity (pro-inflammation from cytokines) **(D)** and **(H)**. The curves for the modes only are shown. Benchmark dose values were calculated from such curves for the mode, lower and upper bounds (2.5 and 97.5%) of the credible intervals (Table 5).

Summary statistics (median and a 95% credible interval) computed from the retained sample from the posterior distribution are given for each of the sensitive parameters in Table 3 **.** To allow for a direct comparison with the prior, similar summary statistics based upon a sample drawn from the prior distribution are also given in Table 3.

**TABLE 3:** Posterior medians and 95% credible intervals for calibrated parameters.

Parameter	Median (95% credible interval)	
	Prior	Posterior
BW	78.5 (46.63, 131.3)	82.19 (56.56, 124.1)
Vmax apical *in vitro*	35,603 (20,793, 61,074)	37,526 (21,393, 54,918)
VfilC	0.0004 (0.0002, 0.0006)	0.00034 (0.00018, 0.00052)
Free	0.0188 (0.0106, 0.031)	0.017 (0.0099, 0.026)
PR	0.11 (0.076, 0.143)	0.121 (0.088, 0.147)
Protein	0.000002 (0.000001, 0.000003)	0.0000021 (0.0000011, 0.0000029)
RAFapi	0.00067 (0.00041, 0.0012)	0.00073 (0.00051, 0.00011)
GFRC	23.10 (14.20, 37.64)	20.73 (13.54, 31.50)
Kurinec	0.06 (0.037, 0.098)	0.0562 (0.0357, 0.088)
PL	1.01 (0.728, 1.40)	1.11 (0.771, 1.42)

A comparison of the two sets of summary statistics illustrates a broad consistency. The medians changed little following calibration; however, there was a consistent narrowing of credible intervals following calibration. The correlations between parameters in the posterior distribution were generally weak, the most notable being those between kurinec and Vmax apical *in vitro* (0.373), protein (0.413), and RAFapi (0.35), respectively.

The fit of the calibrated model to data is shown in Figure 1 was conducted using the same data used in Figures 2–4 in Worley et al. (2017). In the plot, the fit to the three different concentrations is shown and distinguished by color. The solid lines represent the posterior mode fit—this corresponds to the same parameter set for each concentration, with these three simulations only differing in the fixed drinking water concentration. The shaded bands surrounding the posterior modes at each concentration correspond to a numerically derived 95% credible interval: at each time point, the predictions from the retained sample were ordered, and the 2.5 and 97.5 percentiles were read off. This process was repeated for each concentration. The measurements from North Alabama, Lubeck Public Service District, and Little Hocking Water Association, respectively, are shown for comparison and indicate a good overall agreement between the model predictions and measurements. The vertical gray shaded band between 100,000 and 120,000 h, also shown in Figure 1, highlights the point where steady state was judged to have been achieved; the model predictions from this period were used for QIVIVE calculations.

Chemical-specific adjustment factors (CSAFs) of 1.099, 1.098, and 1.096 for the lowest to highest dose curves were calculated from the quotients of the 95th/50th percentiles of the credible intervals shown in Figure 1.

Another potential CSAF would be the quotient of the 95th/50th percentile of the posterior distribution for the organic anion transporters (OATs) in the proximal tubule cells of the kidney. The OATs are thought to be the major determinants of species-dependent differences in biological half-life of PFOA (Nakagawa et al., 2008; Nakagawa et al., 2009). In this study, Vmax apical *in vitro*, the limiting rate of the OAT4 transporter, was identified as one of the ten most sensitive parameters that determine CL and CA. Therefore, a CSAF of 1.4 was calculated by taking the quotient of the 95th/50th percentile of the posterior distribution of Vmax apical *in vitro*.

### Quantitative In Vitro In Vivo Extrapolation

As described in methods, a two-stage approach was used to sample ExposedDW and DWtotal that were consistent with *in vitro* experimental data and a modest degree of model uncertainty, accounted for through accepting simulations within 5% of the target concentration. Results from the first phase (rejection sampling) of the approach are illustrated in Figure 3. Panel A of Figure 3 illustrates concentration response profiles from 500 simulations, whereas panel B shows just the concentration response profiles from the retained simulations that were within 7.5% of the target concentration (of 1,035.175 (µg/L) in this example. In the second phase of analysis an ABC MCMC algorithm was utilized to allow more efficient sampling of the parameter space consistent with a given *in vitro* target concentration. A tighter threshold of 5% was stipulated for the ABC MCMC sampling. This two-stage process was repeated for each *in vitro* concentration. Acceptance rates of between 5 and 20% (median 10%) were achieved for the ABC MCMC simulations. Onward analysis was based upon results from the retained samples and pooled over the four chains run for QIVIVE for each *in vitro* concentration.

The *in vivo* dose responses, Intake (ng/kg BW/day), and ExposedDW (ng/L) estimated from the four *in vitro* concentration–response datasets; estrogen receptor binding (ATG_ERE_CIS_up), pregnane X receptor binding (ATG_PXRE_CIS_up), thyroid hormone receptor binding (ATG_THRa1_TRANS_dn), and plasminogen activator, urokinase receptor protein (BSK_3C_uPAR_down) are provided in Table 4. Posterior distributions for the most sensitive parameters identified by GSA were estimated by updating the prior distributions. So Intake was calculated from ExposedDW, DWtotal, and BW (see Eq. 1), and Table 4 shows results as posterior distributions for the estimates of exposure; Intake and ExposedDW and the parameters; DWtotal and BW required to derive them.

**TABLE 4:** Posterior modes and 97.5% credible ranges for Intake and varying model parameters for target AUC concentrations in liver and serum.

	ATG_ERE_CIS_up			
*In vitro* concentration (µg/L)	Intake (ng/kg BW/Day)	ExposedDW (ng/L)	DWtotal (L)	BW (kg)
1.035	0.19 (0.05, 0.39)	8.79 (4.32, 9.57)	2.96 (1.17, 3.28)	55.72 (42.11, 135.40)
4.141	0.71 (0.21, 1.74)	33.04 (16.66, 34.80)	3.03 (1.31, 3.28))	52.69 (42.10, 134.91)
10.352	1.79 (0.69, 4.32)	78.82 (40.86, 95.24)	2.97 (1.31, 3.29	51.66 (42.14, 135.26)
41.407	4.43 (1.79, 15.21)	322.1 (162.24, 349.83)	2.47 (1.17, 3.27)	60.22 (42.56, 135.60)
103.518	17.0 (4.25, 42.2)	812.16 (430.00, 972.20)	2.71 (1.12, 3.28)	53.09 (43.13, 137.30)
310.553	48.88 (10.67, 123.88)	2,637.03 (1,144.80, 3,001.40)	2.53 (1.08, 3.20)	53.91 (44.15, 134.92)
1,035.175	194.56 (48.00, 409.26)	8,778.20 (4,436.31, 9,708.72)	2.97 (1.24, 3.29)	56.04 (44.13, 138.18)
	**ATG_PXRE_CIS_up**			
1.035	0.15 (0.06, 0.43)	8.87 (4.63, 9.81)	2.60 (1.36, 3.28)	66.61 (43.76, 138.62)
4.141	0.68 (0.30, 1.39)	28.53 (18.46, 36.25)	3.03 (1.40, 3.29)	69.15 (45.57, 142.00)
10.352	1.79 (0.48, 3.87)	77.25 (43.13, 95.67)	2.13 (1.36, 3.28)	63.46 (42.30, 135.74)
41.407	6.06 (2.22, 16.01)	361.50 (199.74, 384.22)	1.76 (1.03, 3.14)	78.17 (42.26, 134.92)
103.518	18.56 (4.30, 42.02)	914.66 (435.73, 985.64)	3.00 (1.23, 3.29)	106.08 (
310.553	57.87 (16.98, 132.37)	2,426.21 (1,272.98, 3,062.36)	2.42 (1.28, 3.27)	54.64 (42.07, 134.30)
1,035.175	181.56 (59.54, 406.70	7,957.00 (4,699.83, 10,119.74)	2.28 (1.27, 3.28)	54.34 (44.85, 140.33)
	**ATG_THRa1_TRANS**			
1.035	0.17 (0.06, 0.36)	6.24 (4.04, 9.54)	3.08 (1.20, 3.29)	56.39 (42.03, 139.84)
4.141	0.56 (0.16, 1.53)	32.51 (16.83, 34.44)	2.77 (1.26, 3.29)	126.29
10.352	7.45 (2.56, 15.89)	85.25 (46.91, 102.19)	2.85 (1.25, 3.29)	76.59 (45.55, 141.37)
41.407	15.76 (5.82, 38.61)	338.90 (181.90, 362.60)	2.94 (1.28, 3.29)	53.82 (42.35, 138.77)
103.518	34.32 (11.02, 116.89)	719.40 (382.52, 932.39)	2.87 (1.50, 3.29)	56.56 (46.75, 142.82)
310.553	1.63 (0.46, 3.88)	2,526.87 (1,052.32, 2,853.18)	2.64 (1.20, 3.29)	135.05 (46.14, 143.13)
1,035.175	144.82 (47.35, 402.22)	8,954.99 (4,266.71, 9,664.36)	2.87 (1.27, 3.29)	125.41 (43.69, 138.62)
	**BSK_3C_uPAR_down**			
9.524	1.73 (0.48, 4.21)	86.35 (35.76, 92.23)	2.58 (1.26, 3.28)	53.95 (42.00, 135.89)
38.094	5.87 (1.93, 13.39)	261.76	2.53 (1.22, 3.29)	56.38 (42.67, 136.80)
95.236	20.73 (5.08, 42.63)	877.62	2.08 (1.33, 3.28)	61.79 (43.28, 136.97)
380.944	59.46 (18.58, 134.05)	2,826.00 (1751.56, 3,592.88)	2.21 (1.25, 3.29)	131.92 (48.43, 143.94)

### Benchmark Dose Analysis

The *in vivo* dose responses provided in Table 4 were used to derive four BMDL_5_ (lower limit of the 95% confidence interval on the benchmark response equivalent to a 5% effect size) for each *in vitro* assay. The mode 2.5 and 97.5% percentile BMDL_5_ values were derived for ExposedDW and Intake by using the mode and upper and lower limits of the credible ranges for each concentration listed in Table 4. The predicted mode for the *in vivo* dose–response curves for each of the *in vitro* datasets for Intake (upper panel) and ExposedDW (lower panel) is shown in Figure 4. The BMDL_5_ values are listed in Table 5 along with the Intake values for serum cholesterol and antibody titer calculated by EFSA.

**TABLE 5:** BMDL5 mode and 95% credible intervals for drinking water exposure concentration and Intake and chemical specific adjustment factors.

	ExposedDW (ng/L)	Intake (ng/kg BW/day)	CSAF (T_½_ Vmax apical *in vitro*)	$\frac{Intake}{CSAF}$	$\frac{Intake}{Default}$	
ATG_ERE_CIS_up	88.3 (163, 304)	6.46 (1.69, 13.8)	1.4	4.61 (0.09–11.1)	0.65 (0.17–1.38)	
ATG_PXRE_CIS_up	139 (129, 323)	0.82 (0.12, 15.6)	1.4	0.59 (0.09–11.1)	0.08 (0.01–1.11)	
ATG_THRa1_TRANS	85.5 (62.5, 107)	3.27 (1.65, 4.82)	1.4	2.34 (1.18–3.44)	0.33 (0.17–0.48)	
BSK_3C_uPAR_down	295 (158, 353)	6.88 (1.98, 14.7)	1.4	4.91 (1.14–10.5)	0.69 (0.20–1.10)	
		EFSA				
		Intake (ng/kg BW/day)		$\frac{vitroBMDL_{5}}{EFSABMDL_{5}}$		
		Mean				
Serum cholesterol (ATG_PXRE_CIS_up)		0.857		0.69		0.09
Decreased antibody titer (BSK_3C_uPAR_down)		1.140		4.31		0.61

The Intake BMDL_5_ mode of 0.82 ng/kg BW/day for pregnane X receptor binding (ATG_PXRE_CIS_up), which may be associated with the perturbation of mitochondrial fatty acid β-oxidation leading to altered serum cholesterol levels, was similar to the BMDL_5_ of 0.86 ng/kg BW/day (6 ng/kg BW/week) for serum cholesterol derived by the EFSA scientific panel on contaminants in the food chain (EFSA Contam Panel et al., 2018). In order to derive a tolerable daily intake for PFOA, the CONTAM panel of EFSA concluded that the application of any additional UFs was not needed because the biological monitoring was based on large epidemiological studies from the general population and performed on risk factors for disease rather than disease endpoints, including potentially sensitive subgroups. Therefore, the BMDL_5_ value as a PoD corresponds to the tolerable daily intake (TDI) for PFOA.

The Intake BMDL_5_ mode of 6.88 ng/kg BW/day for plasminogen activator, urokinase receptor protein (BSK_3C_uPAR_down) which may be associated with pro-inflammation from cytokines and possible altered antibody titer was six-fold higher than the value of 1.14 ng/kg BW/day derived by the EFSA (EFSA Contam Panel, 2020).

Application of the default UF of 10 allowing for inter-individual differences in kinetics and dynamics would reduce the mode to 0.082 for serum cholesterol which is ten-fold lower and 0.69 ng/kg BW/day for antibody titer which is similar to those derived by the EFSA (Table 5).

CSAFs of 1.4 for PFOA allowing for inter-individual variability in kinetics, based on biological half-life which was greater than the CSAF of 1.1 based on AUC (at steady state kinetics) was applied to give an Intake BMDL_5_ of 0.59 for serum cholesterol and 4.91 (ng/kg BW/day) for decreased antibody titer, which were 0.69 and 4.31, respectively, than the EFSA derived values (Table 5).

The BMDL_5_ values of 6.46 and 3.27 ng/kg BW/day for ATG_ERE_CIS_up and ATG_THRa1_TRANS were similar in magnitude to the other datasets although could not be compared with BMDLs used by a regulatory agency in a risk assessment (Table 5).

The corresponding BMDL_5_ modes for ExposedDW, the drinking water concentrations, for ATG_ERE_CIS_up, ATG_PXRE_CIS_up, ATG_THRa1_TRANS, and BSK_3C_uPAR_down were 0.883, 0.139, 0.086, and 0.295 ng/ml, respectively. These concentrations are 5.7-, 36-, 58.5-, and 16.9-fold lower than the measured median level for the general United States population which was reported to be approximately 5 ng/ml (5 μg/L) (Emmett et al., 2006; Steenland et al., 2010).

## Discussion

In this work, we applied the workflow of McNally et al. (2018) in order to account for parameter and model uncertainty within a computationally efficient framework. This the first time the workflow has been applied to a human PBK model and human *in vitro* cell line data. A technical discussion of the framework and in particular a justification for the inclusion of model uncertainty are covered in detail in McNally et al. (2018) and therefore is not repeated here. However, a thorough comparison between the “reverse dosimetry” approach adopted in the workflow and the iterative forward dosimetry “dose matching” approach to QIVIVE that is frequently adopted in the literature is of merit. In our approach, the uncertainties and variabilities in model parameters have been specified through probability distributions, the sensitivity of model output to uncertainties in model inputs has been tested through sensitivity analysis, uncertainty has been refined (although not completely eliminated) through calibration, and finally parameter uncertainties and model uncertainty have been accounted for during QIVIVE. In contrast, the “dose matching” approach does not account for uncertainty at any stage of the modeling process. Results are inevitably sensitive to the default parameters assumed by researchers, but there is currently no mechanism for quantifying this sensitivity.

The PBK model for PFOA was written in MCSim syntax, compiled and subsequently run using the generated executable. MCSim was an appropriate platform given the long simulation period (up to 350,000 h which took around 10 h on the Microsoft Azure IaaS) and the requirement for hundreds of thousands of model evaluations (covering the processes of development and testing, GSA, calibration, and QIVIVE). However, while MCSim is particularly well suited to computationally intensive computations such as MCMC which can be executed at the command line, the command line interface was not well suited to the overall workflow adopted for this work. Interaction with the model executable was undertaken in three distinct ways. RVis was used for development and testing of the model and for GSA. RVis acts as a user-friendly front end to an MCSim executable and supplies input, runs, processes and reports model outputs from the executable. In addition to providing a user-friendly front end for interacting with the model, RVis parallelizes the runs in batch-run operations over the available cores on a personal computer, such that the run time for a computationally expensive technique such as eFAST (GSA), which requires tens of thousands of runs, may be substantially reduced. Calibration was performed using MCMC (a technique for which MCSim is particularly well suited and run through the command line). Model output from MCMC was subsequently processed using R scripts. QIVIVE was also performed using R scripts which supplied inputs to, ran, and processed outputs from the executable. The computation for this process was exported to Azure as described in Methods. At present the full workflow requires significant expertise in PBK modeling, statistics, and programing and is not widely accessible. A focus of current work is to develop a user-friendly module within RVis for QIVIVE, which would substantially reduce the entry barrier to the technique.

This study has demonstrated that the availability of freely accessible *in vitro* concentration–response data for environmental pollutants from the Tox21 and ToxCast high-throughput *in vitro* screening programs could be valuable and effective in chemical risk assessment. The credible intervals for the *in vivo* BMDL values for Intake for serum cholesterol (ATG_PXRE_CIS_up) with and without the application of a CSAF or default UF encompass the BMDL for Intake derived by the EFSA. However, only the *in vivo* BMD values for Intake for decreased antibody titer (BSK_3C_uPAR_down) with application of the CSAF encompassed the value derived and used by the EFSA. These results suggest that the *in vitro* concentration–response data could be used to predict *in vivo* dose response successfully.

At present we address each *in vitro* concentration in turn and estimate corresponding *in vivo* concentrations, accounting for model and parameter value uncertainty with a distribution of *in vivo* concentrations resulting from each *in vitro* concentration. From this data, we derive a dose–response curve from central estimates and calculate a BMD. This BMD can be used in the risk assessment process, with standard uncertainty factors applied as necessary. With a minor change to methodology, our approach could be used to derive CSAFs. The principle change would be to derive the full sequence of *in vivo* concentrations, corresponding to a given set of uncertain parameters and the full sequence of *in vitro* target concentrations, within a single step. The dose–response data could be interpreted as corresponding to a given individual in a population, and a BMD estimated from the data. Through repeating and generating a sample of BMDs, uncertainty factors may be derived. In application, there are considerable technical challenges in achieving a reasonable acceptance rate for proposals and computational efficiency. This is a priority area of research going forward.

However, there are a number of caveats that must be considered. For example, we applied a simple approach to mitigate the use of nominal, applied concentrations. Estimation of the available free concentration of the test compound is a function of serum protein and lipid composition of the media, and it is foreseen that more accurate estimates of bio-actively available *in vitro* concentrations will lead to different and more accurate extrapolated *in vivo* concentrations. In addition, the majority of assay protocols appear to be standardized where the *in vitro* concentrations and spacing are identical for all datasets, spanning three orders of magnitude, ranging from 41.41 to 41,407 μg/L. This may not be ideal for the derivation of a BMDL for which the concentration spacing might need to be altered to more faithfully capture the changes in response. More generally, the assays used in this study involved binding of unmetabolized chemical to a receptor which would initiate a cascade of interactions assumed to cause the perturbation of various biochemical pathways that precede overt toxicity. Therefore, these assays are limited to reactions that do not require metabolism of chemical to an active moiety. Indeed, the challenge of predicting a priori the rate at which a potential toxicant is metabolized *in vivo* still remains unsolved (Wetmore et al., 2012).

It is also important to document known limitations of the assays. For instance, assays using fluorescent readouts can give unreliable results for compounds that are themselves fluorescent (e.g., azo dyes). With cell-based assays, simultaneous cytotoxicity measurements are usually needed because cytotoxicity can confound the target-specific readout. Despite the known limitations of *in vitro* assays, there are plenty of examples of increased utility when combined with PBK models. For example, if *in vivo* clearance is included, approximately two orders of magnitude variation in biological potency could be captured when predicted directly using HTS *in vitro* measurements (Knudsen et al., 2015).

While our ABC algorithm is more appropriate for prioritized chemicals for which a thorough risk assessment is justified, the ability to quantify model parameter, structure, and data uncertainty is of paramount importance for the development of confidence in model use. The ability to do this within an intuitive, freely available software tool would make this approach accessible to a wider user base. This is an important step forward to implement the use of NAMs in chemical risk assessment since QIVIVE and reverse dosimetry have been described as the critical “endgame” in the workflow of predictive toxicology (Knudsen et al., 2015). QIVIVE is essential in order to transition away from animal model–based toxicology to entirely *in vitro*/*in silico*-based toxicological science (Knudsen et al., 2015). It is recommended to further test the current probabilistic workflow allowing the derivation of BMDL from the integration of data from *in vitro* assays, PBK modeling, and QIVIVE approaches, through relevant case studies for chemicals of relevance to the food safety area and chemical risk assessment in general.

## Data Availability

The original contributions presented in the study are included in the article/Supplementary Material; further inquiries can be directed to the corresponding author.

## Footnotes

1 Agency for Toxic Substances and Disease Registry, Atlanta, Georgia 30,341

2
https://www.gnu.org/software/mcsim/

3
https://github.com/GMPtk/MCSimViaRtools

4
http://cefic-lri.org/toolbox/r-vis-open-access-pbpk-modelling-platform/

5
https://github.com/GMPtk/RVis/releases

6
https://proastweb.rivm.nl/

7
https://cran.r-project.org/bin/windows/base/old/3.4.3/

8
https://linuxize.com/post/how-to-install-xrdp-on-ubuntu-18-04/

9
https://comptox.epa.gov/dashboard/

10
https://lri.americanchemistry.com/Users-Guide-for-Accessing-and-Interpreting-ToxCast-Data.pdf

11 Aopwiki.org/aops/200

12 Aopwiki.org/events/451
