# Supplementary material for: Derivation of a Human In Vivo Benchmark Dose for Perfluorooctanoic Acid From ToxCast In Vitro Concentration–Response Data Using a Computational Workflow for Probabilistic Quantitative In Vitro to In Vivo Extrapolation
Source: Front Pharmacol. 2021 May 11;12:630457. doi: 10.3389/fphar.2021.630457 (PMC8144460; doi:10.3389/fphar.2021.630457)
Supplement: Supplementary file 1 [file Table1.docx]

**Appendix: Data from Chemistry Dashboard in original form and transformed for use in ABC algorithm**

| Table S1 | Concentration-response data from Chemistry Dashboard^¶^ | | | | | | | | |
| --- | --- | --- | --- | --- | --- | --- | --- | --- | --- |
| Data from Chemistry Dashboard in original form | | | | | | | | | |
|  | |  | **ATG_PXRE_CIS_up** | **ATG_ERE_CIS_up** | | | **ATG_THRa1_TRANS** | | **BSK_3C_uPAR_down** |
| Nominal in vitro concentration (µM/L) | | **LOG_10_ in vitro Concentration (µM/L)** | **Fold Induction (LOG_2_)** | | | | | | |
| 0.1 | | -1.000 | -0.042 | | 0.019 | | | 0.475 |  |
| 0.4 | | -0.398 | 0.169 | | -0.050 | | | 0.394 |  |
| 1.0 | | 0.000 | 0.151 | | -0.242 | | | 0.003 | 0.084 |
| 4.0 | | 0.602 | 0.027 | | -0.012 | | | -0.345 | 0.092 |
| 10.0 | | 1.000 | 0.178 | | 0.002 | | | 0.443 | 0.153 |
| 30.0 | | 1.477 | 0.811 | | 0.684 | | | 1.334 |  |
| 40.0 | | 1.602 |  | |  | | |  | 0.151 |
| 100.0 | | 2.000 | 1.642 | | 2.029 | | | 2.025 |  |
|  | |  |  | |  | | |  |  |
| Data transformed into natural scale for input into ABC algorithm | | | | | | | | | |
|  | | | | | | | | | |
| Nominal in vitro concentration (µg/L) | | **Estimated free in vitro concentration (µg/L)** | **Fold Induction** | | | | | | |
| 41.4 | | **1.035** | 0.971 | | | 1.013 | | 1.390 |  |
| 165.7 | | **4.141** | 1.124 | | | 0.966 | | 1.314 |  |
| 414.2 | | **10.352** | 1.111 | | | 0.846 | | 1.002 | 1.213 |
| 1656.7 | | **41.407** | 1.019 | | | 0.992 | | 0.787 | 1.236 |
| 4141.7 | | **103.518** | 1.131 | | | 1.002 | | 1.360 | 1.422 |
|  | |  |  | | |  | |  |  |
| 12425.1 | | **310.553** | 1.754 | | | 1.607 | | 2.522 |  |
| 16562.8 | | **380.944** |  | | |  | |  | 1.416 |
| 41417.0 | | **1035.175** | 3.121 | | | 4.082 | | 4.071 |  |

^¶^ <https://comptox.epa.gov/dashboard/dsstoxdb/results?search=DTXSID8031865#invitrodb-bioassays-toxcast-tox21>
